# Association between stigma towards HIV and MSM and intimate partner violence among newly HIV-diagnosed Chinese men who have sex with men

**DOI:** 10.1186/s12889-020-8259-y

**Published:** 2020-02-10

**Authors:** Na Wang, Bo Huang, Yuhua Ruan, K. Rivet Amico, Sten H. Vermund, Shimin Zheng, Han-Zhu Qian

**Affiliations:** 1 School of Public Health, Guilin Medical University, Guilin, China; 2 Guangxi Key Laboratory of AIDS Prevention and Treatment, Nanning, China; 3 State Key Laboratory of Infectious Disease Prevention and Control, Collaborative Innovation Center for Diagnosis and Treatment of Infectious Diseases, Chinese Center for Disease Control and Prevention, Beijing, China; 4 Department of Health Behavior and Health Education, School of Public Health, University of Michigan, Ann Arbor, MI USA; 5 Yale University School of Public Health, New Haven, CT USA; 6 Department of Biostatistics and Epidemiology, College of Public Health, East Tennessee State University, Johnson City, TN USA; 7 SJTU-Yale Joint Center for Biostatistics and Data Science, Shanghai Jiao Tong University (SJTU), Shanghai, China

**Keywords:** HIV-related stigma, MSM-related stigma, Intimate partner violence, Men who have sex with men, HIV

## Abstract

**Background:**

HIV- and MSM-related stigma are well documented as common for Chinese men who have sex with men (MSM) living with HIV, yet there is sparse literature on intimate partner violence (IPV) and its relationship with stigma in this vulnerable population. To evaluate the association between HIV-stigma and stigma related to homosexuality and IPV among newly HIV-diagnosed MSM in China.

**Methods:**

Data were collected in the baseline survey among newly HIV-diagnosed Chinese MSM in a randomized clinical trial via face-to-face interviews. Univariate logistic and multivariate logistic regression analyses were performed to assess the associations between IPV and HIV- and MSM-related stigma.

**Results:**

Of 367 newly HIV-diagnosed Chinese MSM, 23.7% experienced any IPV, including 16.6% physical, 7.4% psychological and 5.2% sexual IPV. Positive associations were found between HIV- and MSM-related stigma and IPV. Men with high HIV-related stigma (score ≥ 27) were 1.67 times as likely to experience any IPV as those with low stigma (adjusted odds ratio [AOR]: 1.67, 95% confidence interval [CI]: 1.02–2.76). Men with high MSM-related stigma (score ≥ 6) were 1.99 times as likely to experience any IPV as those with low stigma (AOR: 1.99, 95% CI: 1.18–3.36).

**Conclusions:**

HIV- and MSM-related stigmas was positively associated with IPV experiences among newly diagnosed MSM in China. The manner in which stigma may exacerbate IPV, and/or the influence of IPV on worsening stigma should be further evaluated. The high prevalence of IPV and stigma in this population suggests that interventions should be taken to reduce stigma and prevent this risky behavior among MSM.

## Background

Intimate partner violence (IPV) generally can occur between current or former spouses, common-law spouses, and nonmarital dating partners, whether of the same opposite sex [1]. Studies of IPV commonly consider physical, sexual, and/or psychological violence in the context of intimate partnership [2]. Men who have sex with men (MSM) in America are more likely to experience IPV during their lifetime than heterosexual men [3, 4]. Globally, prevalence rates among MSM range from 32 to 78% for experiencing any form of IPV [5, 6], 12 to 45% for physical IPV [7, 8], 5 to 33% for sexual IPV [8, 9], and 28 to 64% for emotional/psychological IPV [10, 11]. IPV has been associated with higher levels of potentially risky sexual behaviors, such as condomless sex [5, 12, 13], group sex [14], commercial and casual sex, as well as increased risks for substance abuse [15] and sexually transmitted infection [16].

The experience of IPV may be common among MSM who are HIV-positive in America [9, 12, 17]. HIV-status and related HIV stigma may play an important role in IPV within intimate partnerships among MSM. HIV-related stigma can be defined as the procession of prejudice, discounting, discrediting, and discrimination toward people living with HIV and the individuals, groups, and communities they are connected with [18–20]. HIV stigma can further involve labeling, segregating, stereotyping or discriminating towards people living with HIV via social, economic, or political means [21]. Experiencing HIV-related stigma is not uncommon for MSM living with HIV/AIDS [22, 23], and may play a role in IPV. Among pregnant women living with HIV in South Africa, for example, greater HIV-related stigma was associated with greater combined physical and psychological IPV [24], and women living with HIV reporting high HIV-related stigma reported more frequent physical IPV, social isolation and helplessness [25]. Despite the large number of MSM in China, little is known about the IPV experience among those living with HIV.

In China, stigma concerning HIV can be coupled with stigma concerning same-sex sexual identity. Stigma towards same-sex sexual identity is common in China [26, 27]. Same-sex partnerships challenge the traditional Chinese emphasis on heterosexual child bearing for continuity of family “lines” [28]. Moreover, a person with higher stigma consciousness may be more likely to perceive discrimination towards one’s self [29]. Gay men who in violent relationships may be more likely to keep the intimate partner abuse experience in silence and stay in the violent relationship when stigma consciousness is high [30] .

HIV- and MSM-related stigma [27, 31] are well documented as common for Chinese MSM living with HIV, yet there is sparse literature on IPV and its relationship with stigma in this vulnerable population [16]. We evaluated the prevalence of lifetime IPV and its relationship with HIV- and MSM-related stigma among newly diagnosed HIV-infected MSM in Beijing, China.

## Methods

### Study design and participants

The data for this analysis were from the baseline surveys of a randomized clinical trial called the China MP3 Project. This project had two study phases and was described in detail elsewhere [32]. In brief, men who lived in Beijing, self reported having sex with another man, were 18 years or older and willing to provide written informed consent were recruited (via short message service, website advertisement, gay-frequented venue outreach and peer referral) and tested for HIV in the Phase I and completed a cross-sectional survey. Participants were compensated 30 Chinese yuan (nearly US$15) for completing the survey. Phase I participants who were diagnosed with HIV were invited to participate in the Phase II randomized intervention clinical trial (RCT) evaluating the efficacy of text messaging and peer-led counseling on linkage to HIV care and initiation of antiretroviral therapy. Phase II participants were compensated 100 Yuan (about 15 dollars) per study survey completion. A total of 367 newly diagnosed MSM consented and were enrolled in the Phase II RCT. Data from both Phase I cross-sectional survey and Phase II baseline survey were used in this analysis.

This study was approved by the institutional review boards of the National Center for AIDS/STD Control and Prevention of the Chinese Center for Disease Control (No. X120331206) and Prevention and Vanderbilt University with the number (IRB# 111144).

### Data collection and measurement

Sociodemographic and behavioral data, including age, ethnicity, marital status, current living status, education, employment, health insurance plan, personal monthly income, place of birth, registered Beijing household (or *Hukou*), length of residence in Beijing, illicit drug and alcohol use, IPV, and HIV- and MSM-related stigma were collected through face-to-face interviews by trained clinical nurse. Drug use was assessed as type of substance used (ever) and a follow-up for any substance used for frequency of use in past 3-months (never to 4 or more times a week). For alcohol use, participants were asked the item on frequency of use. Participants were then categorized for use in the past 3 months at ever versus (vs) never use.

HIV-related stigma was measured with Steward’s HIV stigma scale, which includes four subscales: enacted stigma (e.g., “Have you been refused housing because people suspect you have HIV?”); felt stigma (e.g., “How many people think people with HIV are paying for their karma or sins?”); vicarious stigma (e.g., “A village/community ostracizes someone because they have HIV?”); and internalized stigma (e.g., “that you are paying for karma or sins because you have HIV?”) [33]. In the present study, 37% of 367 newly HIV-diagnosed Chinese MSM did not respond to five or more of 10 enacted stigma items. Thus, the enacted stigma items were disregarded, and the remaining three subscales were included in the data analysis. Cronbach’s alpha values were 0.97, 0.92 and 0.94 for felt, vicarious, and internalized stigma, respectively.

Each subscale contains 10 items, and each item was scored from 0 (“never” or “no one” or “not at all” ) to 3 (“frequently” or “most people” or “a great deal”), with Total possible scores thus ranged from 0 to 90, with higher scores indicative of more HIV-related stigma we created a dichotomous variable of below vs. at or above the mean. For dichotomous representation of this variable, we defined high as at or above the sample mean.

MSM-related stigma was measured with Neilands’ homosexual stigma scale [34]. This scale was initially published in English by Diaz and was translated into Mandarin [35]. Its Chinese version further reviewed by two study team members who are fluent in both Chinese and English. This scale consists of perceived stigma (e.g., “How often have you felt that your homosexuality hurt and embarrassed your family?”) and enacted stigma (e.g., “How often have you lost your friends because of your homosexuality”).

This scale contains 10 items, each scoring from 0 (“*never*”), 1 (“*once or twice*), 2 (“*a few times”*), to 3 (“*many times”*). Cronbach’s alpha values were 0.71, 0.81 and 0.76 for perceived, enacted and total MSM-related stigma, respectively. The total score of MSM-related stigma ranges from 0 to 30, with higher scores reflecting more MSM-related stigma. For analyses, we created a dichotomous variable of below vs. above the mean.

Physical IPV experience was defined as ever being in an intimate relationship with a person who physically hurt the participant, including pushing, hitting you, holding you down, attempting to strangle or attacking with a weapon. Psychological IPV experience was defined as ever being in an intimate relationship with a person who imparted threats, insults, maltreatments, or fears. Sexual IPV experience was defined as ever being in an intimate relationship with a person who forced to have sexual activities that were uncomfortable or undesired. Any IPV was defined as any one of the above. All IPV variables were dichotomous (ever vs. never).

### Statistical analysis

The primary dependent variable was any IPV. The main predictors of any IPV were HIV-related stigma and MSM-related stigma. HIV-related stigma and MSM-related stigma were each classified into two categories: high if a score is greater than or equal to the mean and low if the score is less than the mean [36, 37]. Simple descriptive statistics (mean, standard deviation [SD], proportion) of the main outcome variables and predictors were calculated. Chi-square tests were used to evaluate factors associated with IPV, HIV-related stigma, and MSM-related stigma in univariate analyses. Univariate logistic regression was used to assess the association between HIV-related stigma and MSM-related stigma and IPV. Multivariate logistic regression was performed to evaluate the association while adjusted for other factors including age, marital status, health insurance and place of birth. Considering the possible interaction between HIV-related stigma and MSM-related stigma, we further ran the model with an interaction term to see if there is an interaction. All analyses were performed using SAS (SAS 9.4, SAS Institute, Inc., Cary, NC).

## Results

### Demographic and behavioral characteristics

Of 367 newly HIV-diagnosed Chinese MSM participants, the majority were ≤ 30 years old (65%), Han ethnic (93%), single (88%), college educated (77%), employed (83%), born in an urban region (71%), and migrants (82%). About half had health insurance (55%). In the past 3 months, half reported alcohol use (55%) and one third reported illicit drugs use (33%).

**Table 1:** Sociodemographic and behavioral associates of any IPV and stigma among 367 participants

Factors	Any IPV *N* (%)	*P*	HIV-related stigma(≥27) *N* (%)	*P*	MSM- related stigma (≥6) *N* (%)	*P*
Age, year		0.24		0.83		0.04
≤ 30	61 (25.6)		119 (50.0)		125 (52.5)	
> 30	26 (20.2)		66 (51.2)		82 (63.6)	
Ethics		0.97		0.51		0.71
Han	81 (23.7)		174 (50.9)		192 (56.1)	
Other	6 (24.0)		11 (44.0)		15 (60.0)	
Marital status		0.59		0.05		0.56
Single	78 (24.2)		169 (52.3)		184 (57.0)	
Currently married	9 (20.5)		16 (36.4)		23 (52.3)	
Currently living with		0.83		0.58		0.36
Male sexual partner	18 (22.8)		42 (53.2)		41 (51.9)	
Others	69 (24.0)		143 (49.7)		166 (57.6)	
Education		0.12		0.47		0.96
< High school	15 (30.0)		23 (46.0)		29 (58.0)	
High school	12 (34.3)		15 (42.9)		20 (57.1)	
College	60 (21.3)		147 (52.1)		158 (56.0)	
Employment		0.38		0.97		0.11
Employed	72 (23.7)		154 (50.7)		178 (58.6)	
Unemployed	7 (18.0)		19 (48.7)		16 (41.0)	
Student	8 (33.3)		12 (50.0)		13 (54.2)	
Personal monthly income, Chinese yuan		0.14		0.54		0.19
< 5000	58 (26.4)		108 (49.1)		118 (53.6)	
≥ 5000	29 (19.7)		77 (52.4)		89 (60.5)	
Have a health insurance plan		0.04		0.58		0.60
Yes	40 (19.7)		105 (51.7)		117 (57.6)	
No	47 (28.7)		80 (48.8)		90 (54.9)	
Place of birth		0.52		0.20		< 0.01
Urban	59 (22.8)		125 (48.3)		133 (51.4)	
Rural	28 (25.9)		60 (55.6)		74 (68.5)	
Registered Beijing household (*Hukou*)		0.91		0.31		0.95
Yes	16 (24.2)		37 (56.1)		37 (56.1)	
No	71 (23.6)		148 (49.2)		170 (56.5)	
Time living in Beijing, year		0.15		0.43		0.11
< 5	49 (26.9)		88 (48.4)		95 (52.2)	
≥ 5	38 (20.5)		97 (52.4)		112 (60.5)	
Drinking alcohol in the past 3 months						
Yes	46 (22.8)	0.64	104 (51.5)	0.65	121 (59.9)	0.13
No	41 (24.9)		81 (49.1)		86 (52.1)	
Using illicit drugs in the past 3 months						
Yes	26 (21.5)	0.48	65 (53.7)	0.37	73 (60.3)	0.29
No	61 (24.8)		120 (48.8)		134 (54.5)	

Note: *IPV* Intimate partner violence, *MSM* Men who have sex with men

The mean score of HIV-related stigma among the study population was 26.9 (standard deviation [SD]: 20.9), 14.7 (SD: 11.2) for felt stigma, 4.9 (SD: 6.5) for vicarious stigma, and 8.1 (SD: 9.0) for internalized stigma, respectively. The mean score of MSM-related stigma for participants were 6.1(SD: 4.3), 4.6 (SD: 2.8) for perceived stigma, and 1.02 (SD: 2.1) for enacted stigma, respectively. Participants who experienced IPV were less likely to have health insurance. Single men were more likely to have a higher HIV-related stigma (score ≥ 27). Younger age (≤30 year) and being born in the countryside were associated with a ‘high’ MSM-related stigma score (≥ 6) (Table 1).

### Prevalence of intimate partner violence and HIV- and MSM-related stigma

Nearly a quarter (23.7%) of participants reported ever experienced IPV. The most common type of IPV was physical IPV (16.6%) and followed by psychological (7.4%) and sexual (5.2%). Nearly half (50.4%) of participants were categized in the ‘high HIV-related stigma’ group (score ≥ 27), 53.1, 35.7 and 42.0% in the ‘high felt stigma’ group (≥15), ‘high vicarious stigma’ group (≥5) and ‘high internalized stigma’ group (≥8), separately. Totally 56.4% had high MSM-related stigma (score ≥ 6), 53.7% had high perceived stigma (≥5), 33.8% had high enacted stigma (≥1) (Table 2).

**Table 2:** HIV- and MSM-related stigma by IPV type among 367 participants

Stigma	Participants	Any IPV		Physical IPV		Psychological IPV		Sexual IPV	
	N(%)	N(%)	*P*	N(%)	*P*	N(%)	*P*	N(%)	*P*
HIV-related stigma			0.05		0.36		0.18		0.11
< 27	182 (49.6)	35 (19.2)		27 (14.8)		10 (5.5)		6 (3.3)	
≥ 27	185 (50.4)	52 (28.1)		34 (18.4)		17 (9.2)		13 (7.0)	
Felt stigma			0.24		0.66		0.29		0.17
< 15	172 (46.9)	36 (20.9)		27 (15.7)		10 (5.8)		6 (3.5)	
≥ 15	195 (53.1)	51 (26.2)		34 (17.4)		17 (8.7)		13 (6.7)	
Vicarious stigma			0.13		0.95		0.07		0.04
< 5	236 (64.3)	50 (21.2)		39 (16.5)		13 (5.5)		8 (3.4)	
≥ 5	131 (35.7)	37 (28.2)		22 (16.8)		14 (10.7)		11 (8.4)	
Internalized stigma			0.11		0.12		0.79		0.33
< 8	213 (58.0)	44 (20.7)		30 (14.1)		15 (7.0)		9 (4.2)	
≥ 8	154 (42.0)	43 (27.9)		31 (20.1)		12 (7.8)		10 (6.5)	
MSM-related stigma			0.01		0.11		0.05		0.28
< 6	160 (43.6)	28 (17.5)		21 (13.1)		7 (4.4)		6 (3.8)	
≥ 6	207 (56.4)	59 (28.5)		40 (19.3)		20 (9.7)		13 (6.3)	
Perceived stigma(vs.)			0.19		0.14		0.55		0.71
< 5	170 (46.3)	35 (20.6)		23 (13.5)		11 (6.5)		8 (4.7)	
≥ 5	197 (53.7)	52 (26.4)		38 (19.3)		16 (8.1)		11 (5.6)	
Enacted stigma			< 0.01		0.32		< 0.01		< 0.01
< 1	243 (66.2)	46 (18.9)		37 (15.2)		10 (4.1)		6 (2.5)	
≥ 1	124 (33.8)	41 (33.1)		24 (19.4)		17 (13.7)		13 (10.5)	

Note: *IPV* Intimate partner violence, *MSM* Men who have sex with men

Crude analyses suggested that any IPV experiences including physical, psychological and sexual were higher among participants with high HIV- and MSM-related stigma than among those with low stigma, but the differences were statistically significant only for any IPV and not for any individual types of IPV. For the subgroups of HIV- and MSM related stigma, sexual IPV experience was higher among participants with higher vicarious HIV-related stigma and enacted MSM-related stigma, and psychological IPV and any IPV experiences were higher among participants with higher enacted MSM-related stigma (Table 2).

### Associations between HIV- and MSM-related stigma and any IPV experience

The interaction between HIV-related stigma and MSM-related stigma terms in the multiple variate modeling was not statistically significant. Both HIV- and MSM-related stigmas were positively associated with any IPV in univariate logistic regression analyses. After controlling for age, marital status, health insurance and place of birth, high HIV-related stigma (score ≥ 27) was associated with a 67% increase in the odds of any IPV experience (adjusted OR [AOR]: 1.67, 95% confidence interval [CI]: 1.02–2.76); high MSM-related stigma (score ≥ 6) was associated with double odds of any IPV experience (AOR: 1.99, 95% CI: 1.18–3.36). In subgroup analysis, higher enacted MSM-related stigma was associated with double odds of any IPV experience (AOR: 2.13, 95% CI: 1.29–3.50) (Table 3);

**Table 3:** Associations between HIV- and MSM-related stigma and any IPV among 367 participants

Covariate	Any IPV	
	Crude OR (95% CI)	AOR(95% CI)
HIV-related stigma (< 27 vs. ≥27)	1.64 (1.01–2.68)*	1.67 (1.02–2.76)*
Felt stigma (< 15 vs. ≥15)	1.34 (0.82–2.18)	1.38 (0.84–2.27)
Vicarious stigma (< 5 vs. ≥5)	1.46 (0.90–2.40)	1.50 (0.91–2.47)
Internalized stigma (< 8 vs. ≥8)	1.49 (0.92–2.41)	1.53 (0.93–2.51)
MSM-related stigma (< 6 vs. ≥6)	1.88 (1.13–3.12)*	1.99 (1.18–3.36)**
Perceived stigma (< 5 vs. ≥5)	1.38 (0.85–2.25)	1.46 (0.89–2.39)
Enacted stigma (< 1 vs. ≥1)	2.12 (1.29–3.46)**	2.13 (1.29–3.50)**
HIV-related stigma*MSM-related stigma	0.56 (0.20–1.59)	0.54 (0.19–1.54)

Note: *IPV* Intimate partner violence, *OR* Odds ratio, *AOR* Adjusted odds ratio (adjusted for age, marital status, health insurance and place of birth); *CI* Confidence interval, *MSM* Men who have sex with men

^*^Significant at *P* < 0.05; ^**^Significant at *P* < 0.01

## Discussion

In this study, we found that there was a significant association between any IPV experience and stigma among newly HIV-diagnosed Chinese MSM. Higher levels of both HIV- and MSM-related stigma indicated significantly increased odds of reporting any form of IPV after adjusted for potential confounders. Higher level of HIV-related stigma may play a role in social isolation and helplessness among victims of IPV [25]. Sexual minority MSM who are acutely aware of stigma (high stigma consciousness) may fear discrimination and negative treatment from others in the community, limiting accessing resources or seeking outside help [30]. Previous studies have identified the relationship between IPV and internalized homophobia, homophobic discrimination among MSM [38, 39], European American lesbian and bisexual women [40], gay and lesbian [30], and gay, bisexual, transgender, and queer people [41]. The association between stigma and IPV also can be elucidated since stigma consciousness is positively correlated with depression [42, 43], which is strongly associated with IPV [5, 44–47]. Individuals who experience IPV may report internalized stigma as the most common source of stigma. Anticipated stigma may occur when they expect to be negatively judged or devalued if they disclose IPV experiences to others [48]. It remains unclear whether IPV precedes or follows stigma or if the two co-occur, creating environments that perpetuate a cycle of isolation and abuse. Further mixed methods research could provide critical information about the patterning of these experiences with MSM living with HIV.

The observed prevalence of any IPV (23.7%) in our study is in accordance with the rates among Chinese MSM in other studies (24–29%) [14, 16], but is higher than general males (7.6%) [49]. The prevalence of physical IPV experience in our study participants is higher than that among MSM in a Shanghai study (16.6% vs. 6.6%), whereas the rates of psychological IPV (7.4% vs. 8.2%) and sexual IPV (5.2% vs. 5.5%) were nearly the same [16]. The primary limitation of this study is the nature of cross-sectional surveys, which precludes assessing the temporal relationship between stigma and IPV. The association between HIV- and MSM-related stigma and IPV experience is likely bidirectional: maybe MSM with HIV- and MSM-related stigma are more likely to experience IPV, and it is also possible that IPV experience makes MSM more concerned about stigma. Either way, further research – preferably longitudinal in nature - is needed to better characterize these phenomena. Second, our study was conducted among newly HIV-diagnosed MSM in one city, and the study conclusion may have limited generalizability among MSM in other parts of China. Data from multi-center large sample study may provide more generalizable evidence. Third, we investigated the IPV experience through a simple item questionnaire and did not investigate IPV perpetration. More comprehensive and refined future studies with multiple items measurement are needed. Furthermore, only 50% of the participants responded to all of the 10 enacted stigma items [43], and enacted stigma was not included in any HIV-related stigma in our study.

Despite the limitations, this study has strengths. Of importance, we found a correlation between HIV- and MSM-related stigma and IPV among newly HIV-diagnosed MSM, who have been largely ignored. We also measured the prevalence of physical, psychological, sexual, and any IPV. The findings on specific types of IPV may be helpful for guiding the development of intervention programs. This study fills in the knowledge gap in this population.

There is no government program in China to prevent IPV among MSM and other HIV high risk groups. Our study suggested that IPV is common and it is positively associated with HIV- and MSM-stigma, HIV prevention intervention programs among MSM should provide information about warning signs for, or consequences of, intimate partner and sexual violence; these programs may also incorporate strategies to teach healthy relationship skills, promote social norms that protect against violence, and create protective environments. Professional counseling on reduction of stigma and prevention of IPV and supports from family and community should be available for those in need.

## Conclusions

Our study found a relationship between HIV- and MSM-related stigma and IPV among newly HIV-diagnosed Chinese MSM. Further studies are needed to elucidate the reasons for and patterning of this relationship. The high prevalence of IPV and stigma in this population suggests that interventions should be taken to reduce stigma and prevent this risky behavior among MSM.

## Data Availability

The primary data used in the current study will be available from https://rocket.app.vumc.org/index.php?wg=ws5117.

## Abbreviations

AIDS: Acquired immunodeficiency syndrome
AOR: Adjusted odds ratio
CI: Confidence interval
HIV: Human immunodeficiency virus
IPV: Intimate partner violence
MSM: Man who has sex with other men
OR: Odds ratio
RCT: Randomized intervention clinical trial

## References

[1] Chang JC, Cluss PA, Ranieri L, Hawker L, Buranosky R, Dado D (2005). Health care interventions for intimate partner violence: what women want. Women's Health Issues.

[2] Duvvury N, Callan A, Carney P, Raghavendra S. Intimate Partner Violence: Economic Costs and Implications for Growth and Development. World Bank; 2014. https://openknowledge.worldbank.org/handle/10986/16697. Accessed 2013.

[3] Blosnich JR, Bossarte RM (2009). Comparisons of intimate partner violence among partners in same-sex and opposite-sex relationships in the United States. Am J Public Health.

[4] Messinger AM (2011). Invisible victims: same-sex IPV in the National Violence against Women Survey. J Interpers Violence.

[5] Houston E, McKirnan DJ (2007). Intimate partner abuse among gay and bisexual men: risk correlates and health outcomes. J Urban Health.

[6] Pantalone DW, Schneider KL, Valentine SE, Simoni JM (2012). Investigating partner abuse among HIV-positive men who have sex with men. AIDS Behav.

[7] Stephenson R, Khosropour C, Sullivan P (2010). Reporting of intimate partner violence among men who have sex with men in an online survey. West J Emerg Med.

[8] Craft SM, Serovich JM (2005). Family-of-origin factors and partner violence in the intimate relationships of gay men who are HIV positive. J Interpers Violence.

[9] Greenwood GL, Relf MV, Huang B, Pollack LM, Canchola JA, Catania JA (2002). Battering victimization among a probability-based sample of men who have sex with men. Am J Public Health.

[10] Pruitt KL, White D, Mitchell JW, Stephenson R (2015). Sexual agreements and intimate-partner violence among male couples. Int J Sex Health.

[11] Bartholomew K, Regan KV, White MA, Oram D (2008). Patterns of abuse in male same-sex relationships. Violence Vict.

[12] Kalichman S, Benotsch E, Rompa D, Gore-Felton C, Austin J, Luke W (2001). Unwanted sexual experiences and sexual risks in gay and bisexual men: associations among revictimization, substance use, and psychiatric symptoms. J Sex Res.

[13] Raj A, Santana MC, La Marche A, Amaro H, Cranston K, Silverman JG (2006). Perpetration of intimate partner violence associated with sexual risk behaviors among young adult men. Am J Public Health.

[14] Davis A, Best J, Wei C, Luo J, Van Der Pol B, Meyerson B (2015). Intimate partner violence and correlates with risk behaviors and HIV/STI diagnoses among men who have sex with men and men who have sex with men and women in China: a hidden epidemic. Sex Transm Dis.

[15] Decker MR, Miller E, McCauley HL, Tancredi DJ, Anderson H, Levenson RR (2014). Recent partner violence and sexual and drug-related STI/HIV risk among adolescent and young adult women attending family planning clinics. Sex Transm Infect.

[16] Liu Y, Zhang Y, Ning Z, Zheng H, Ding Y, Gao M (2018). Intimate partner violence victimization and HIV infection among men who have sex with men in Shanghai, China. Biosci Trends.

[17] Stall R, Mills TC, Williamson J, Hart T, Greenwood G, Paul J (2003). Association of co-occurring psychosocial health problems and increased vulnerability to HIV/AIDS among urban men who have sex with men. Am J Public Health.

[18] Herek GM (2002). Thinking about AIDS and stigma: a psychologist's perspective. J Law Med Ethics.

[19] Herek GM, Mitnick L, Burris S, Chesney M, Devine P, Fullilove MT (1998). Workshop report: AIDS and stigma: a conceptual framework and research agenda. AIDS Public Policy J.

[20] Malave S, Ramakrishna J, Heylen E, Bharat S, Ekstrand ML (2014). Differences in testing, stigma, and perceived consequences of stigmatization among heterosexual men and women living with HIV in Bengaluru, India. AIDS Care.

[21] Mahajan AP, Sayles JN, Patel VA, Remien RH, Sawires SR, Ortiz DJ (2008). Stigma in the HIV/AIDS epidemic: a review of the literature and recommendations for the way forward. AIDS (London, England).

[22] Hibbert M, Crenna-Jennings W, Kirwan P, Benton L, Lut I, Okala S (2018). The people living with HIV stigma survey UK 2015: HIV-related sexual rejection and other experiences of stigma and discrimination among gay and heterosexual men. AIDS Care.

[23] Burnham KE, Cruess DG, Kalichman MO, Grebler T, Cherry C, Kalichman SC (2016). Trauma symptoms, internalized stigma, social support, and sexual risk behavior among HIV-positive gay and bisexual MSM who have sought sex partners online. AIDS Care.

[24] Matseke G, Rodriguez VJ, Peltzer K, Jones D (2016). Intimate partner violence among HIV positive pregnant women in South Africa. J Psychol Afr.

[25] Fiorentino Marion, Sagaon-Teyssier Luis, Ndiaye Khadim, Suzan-Monti Marie, Mengue Marie-Thérèse, Vidal Laurent, Kuaban Christopher, March Laura, Laurent Christian, Spire Bruno, Boyer Sylvie (2019). Intimate partner violence against HIV-positive Cameroonian women: Prevalence, associated factors and relationship with antiretroviral therapy discontinuity—results from the ANRS-12288 EVOLCam survey. Women's Health.

[26] Zhu Y, Liu J, Chen Y, Zhang R, Qu B (2018). The relation between mental health, homosexual stigma, childhood abuse, community engagement, and unprotected anal intercourse among MSM in China. Sci Rep.

[27] Wei C, Cheung DH, Yan H, Li J, Shi LE, Raymond HF (2016). The Impact of Homophobia and HIV Stigma on HIV Testing Uptake Among Chinese Men Who Have Sex With Men: a Mediation Analysis. J Acquir Immune Defic Syndr.

[28] Choi KH, Hudes ES, Steward WT (2008). Social discrimination, concurrent sexual partnerships, and HIV risk among men who have sex with men in Shanghai, China. AIDS Behav.

[29] Pinel EC (1999). Stigma consciousness: the psychological legacy of social stereotypes. J Pers Soc Psychol.

[30] Carvalho AF, Lewis RJ, Derlega VJ, Winstead BA, Viggiano C (2011). Internalized sexual minority stressors and same-sex intimate partner violence. J Fam Violence.

[31] Dowshen N, Binns HJ, Garofalo R (2009). Experiences of HIV-related stigma among young men who have sex with men. AIDS Patient Care STDS.

[32] Liu Y, Vermund SH, Ruan Y, Liu H, Rivet Amico K, Simoni JM (2018). Peer counselling versus standard-of-care on reducing high-risk behaviours among newly diagnosed HIV-positive men who have sex with men in Beijing, China: a randomized intervention study. J Int AIDS Soc.

[33] Steward WT, Herek GM, Ramakrishna J, Bharat S, Chandy S, Wrubel J (2008). HIV-related stigma: adapting a theoretical framework for use in India. Soc Sci Med.

[34] Neilands TB, Steward WT, Choi KH (2008). Assessment of stigma towards homosexuality in China: a study of men who have sex with men. Arch Sex Behav.

[35] Diaz RM, Ayala G, Bein E, Henne J, Marin BV (2001). The impact of homophobia, poverty, and racism on the mental health of gay and bisexual Latino men: findings from 3 US cities. Am J Public Health.

[36] Wang N, Wang S, Qian HZ, Ruan Y, Amico KR, Vermund SH (2019). Negative associations between general self-efficacy and anxiety/depression among newly HIV-diagnosed men who have sex with men in Beijing, China. AIDS Care.

[37] Adewuya AO, Adeyeye OO (2017). Anxiety and depression among Nigerian patients with asthma; association with sociodemographic, clinical, and personality factors. J Asthma.

[38] Stephenson R, Finneran C (2017). Minority stress and intimate partner violence among gay and bisexual men in Atlanta. Am J Mens Health.

[39] Finneran C, Stephenson R (2014). Intimate partner violence, minority stress, and sexual risk-taking among U.S. men who have sex with men. J Homosex.

[40] Balsam KF, Szymanski DM (2005). Relationship quality and domestic violence in women’s same-sex relationships: the role of minority stress. Psychol Women Q.

[41] Edwards KM, Sylaska KM (2013). The perpetration of intimate partner violence among LGBTQ college youth: the role of minority stress. J Youth Adolesc.

[42] Lewis RJ, Derlega VJ, Griffin J, Krowinski A (2003). Stressors for gay men and lesbians: life stress, gay-related stress, stigma consciousness, and depressive symptoms. J Soc Clin Psychol.

[43] Tao J, Wang L, Kipp AM, Qian HZ, Yin L, Ruan Y (2017). Relationship of stigma and depression among newly HIV-diagnosed Chinese men who have sex with men. AIDS Behav.

[44] Graham K, Bernards S, Flynn A, Tremblay PF, Wells S (2012). Does the relationship between depression and intimate partner aggression vary by gender, victim-perpetrator role, and aggression severity?. Violence Vict.

[45] Beydoun HA, Beydoun MA, Kaufman JS, Lo B, Zonderman AB (2012). Intimate partner violence against adult women and its association with major depressive disorder, depressive symptoms and postpartum depression: a systematic review and meta-analysis. Soc Sci Med.

[46] Siemieniuk RA, Miller P, Woodman K, Ko K, Krentz HB, Gill MJ (2013). Prevalence, clinical associations, and impact of intimate partner violence among HIV-infected gay and bisexual men: a population-based study. HIV Med.

[47] Mays VM, Cochran SD (2001). Mental health correlates of perceived discrimination among lesbian, gay, and bisexual adults in the United States. Am J Public Health.

[48] Murray CE, Crowe A, Overstreet NM (2018). Sources and components of stigma experienced by survivors of intimate partner violence. J Interpers Violence.

[49] Tjaden P, Thoennes N. Extent, nature, and consequences of intimate partner violence: Findings from the National Violence Against Women Survey. 2000. http://www.ncjrsgov/pdffiles1/nij/181867pdf. Accessed 2000.

